# N6-methyladenosine mRNA methylation is important for the light response in soybean

**DOI:** 10.3389/fpls.2023.1153840

**Published:** 2023-04-04

**Authors:** Liya Zhang, Yanyan Zhang, Jun Liu, Hongyu Li, Bin Liu, Tao Zhao

**Affiliations:** The National Key Facility for Crop Gene Resources and Genetic Improvement (NFCRI), Institute of Crop Science, Chinese Academy of Agricultural Sciences, Beijing, China

**Keywords:** soybean, M6A, light response, signaling pathway, epitranscriptomic modification

## Abstract

N6-methyladenosine (m^6^A) modification of messenger RNA (mRNA) is the most prevalent and abundant modification in eukaryotic mRNA and posttranscriptionally modulates the transcriptome at almost all stages of mRNA metabolism. In plants, m^6^A is crucial for embryonic-phase growth, flowering time control, microspore generation and fruit maturation. However, the role of m^6^A in plant responses to light, the most important environmental stimulus, remains unexplored. Here, we profile the m^6^A transcriptome of Williams 82, a soybean cultivar, and reveal that m^6^A is highly conserved and plays an important role in the response to light stimuli in soybean. Similar to the case in *Arabidopsis*, m^6^A in soybean is enriched not only around the stop codon and within the 3’UTR but also around the start codon. Moreover, genes with methylation occurring in the 3’UTR have higher expression levels and are more prone to alternative splicing. The core genes in the light signaling pathway, *GmSPA1a*, *GmPRR5e* and *GmBIC2b*, undergo changes in methylation modification and transcription levels in response to light. KEGG pathway analysis revealed that differentially expressed genes with differential m^6^A peaks were involved in the “photosynthesis” and “circadian rhythm” pathways. Our results highlight the important role played by epitranscriptomic mRNA methylation in the light response in soybean and provide a solid basis for determining the functional role of light on RNA m^6^A modification in this plant.

## 1 Introduction

More than 150 RNA modifications have been identified on various types of RNAs, including mRNA, tRNA, rRNA, lncRNA, and microRNA (Cantara et al., 2011; Meyer and Jaffrey, 2014; Boccaletto et al., 2018). Common mRNA modifications include N^6^-methyladenosine (m^6^A), 5-methylcytidine (m^5^C), N^1^-methyladenosine (m^1^A), and pseudouridine (Ψ) (Roundtree et al., 2017). m^6^A is the most abundant methylation modification in eukaryotic mRNA, and it is also the most thoroughly studied type of RNA modification (Shi et al., 2017). m^6^A methylation is a reversible process that is dynamically coordinated by writers, readers and erasers. RNA m^6^A methylation is a unique molecular mechanism for influencing gene expression (Meyer and Jaffrey, 2014). Studies have shown that m^6^A plays a functional role in mRNA, altering properties such as stability (Frye et al., 2018), decay (Yang et al., 2018), splicing (Haussmann et al., 2016; Xiao et al., 2016), translation efficiency (Meyer et al., 2015; Luo et al., 2020), nuclear export (Zheng et al., 2013) and localization (Hartmann et al., 1999; Yang et al., 2018).

In plants and other eukaryotes, m^6^A modification occurs at a highly conserved RRACH (R = G or A; H: U>A>C) motif (Shen et al., 2016). Unlike that in animals, m^6^A methylation modification in plants is present not only in stop codons and 3’untranslated regions (3’UTR) (Dominissini et al., 2012; Meyer et al., 2012; Schwartz et al., 2014; Ke et al., 2015), but also in start codons (Slobodin et al., 2017). m^6^A modification can affect many aspects of plant biology, including embryo development, shoot apical meristem development, apical dominance, organ size, root development, viral infection, floral transition, leaf and trichome morphogenesis, flowering time, microspore generation and fruit ripening (Shen et al., 2019). There is increasing evidence that m^6^A is also involved in regulating responses to environmental stresses (Wei et al., 2018; Yue et al., 2019; Huong et al., 2020). For example, in *Arabidopsis*, m^6^A methylation plays an important role in salt stress tolerance (Hu et al., 2021). The m^6^A peaks in Pak-choi and rice under heat stress and cadmium stress were significantly reduced compared with those in controls (Liu et al., 2020; Cheng et al., 2021). The methyltransferase *PtrMTA* enhances drought tolerance by regulating the development of trichomes and roots (Lu et al., 2020). Disruption of RNA demethylase AtALKBH10B resulted in the suppression of heat-activated genes and greatly reduced plant fertility (Wang et al., 2022). *AtMTA* can regulate the methylation level and translation efficiency of *DGAT1*, which preliminarily revealed the molecular mechanism of mRNA m^6^A modification in chilling responses in *Arabidopsis* (Wang et al., 2023). In addition, Under high light conditions, m^6^A writer VIRILIZER (VIR) positively regulates photosynthesis, and its mutation significantly reduces photosynthetic activity and photosystem protein abundance (Zhang et al., 2022). These findings clearly indicate that m^6^A is essential for plant growth and development. However, the significance and cellular role of m^6^A RNA modification in the plant response to environmental stimuli remain largely unknown.

Light is essential for plants, and perception of the light environment determines plant growth, morphology, and developmental changes. Light acts as an important developmental switch, affecting photomorphogenesis, germination, flowering time and circadian rhythms (Hoecker, 2017). Soybean is a typical short-day plant. Compared with other crops, soybean is particularly dependent on specific light conditions for its growth and development, which restricts soybean cultivation to particular areas (Li et al., 2020). Its yield at a given latitude depends mainly on the appropriate photoperiod (Lu et al., 2017). However, it is possible to genetically alter soybeans to reduce this photoperiod sensitivity, creating strains that can be grown in a wide range of locations and agricultural settings, to increase yields. Core genes in the light signaling pathway, such as Suppressor of phytochrome A (SPA), Blue light inhibitors of cryptochromes (BIC) and *Pseudoresponse regulator 5* (PRR5), sense changes in the light environment, which in turn affects plant growth and development (Wang et al., 2017; Hwang et al., 2021; Ponnu and Hoecker, 2021). For example, GmBICs regulate soybean stem elongation under low levels of blue light (Mu et al., 2022). The *Arabidopsis* COP1/SPA complex acts as a cullin4-based E3 ubiquitin ligase to suppress photomorphogenesis in darkness (Kerner et al., 2021). PRR5 is one of the core circadian clock components, and *PRR5* mutants in *Arabidopsis* display a shorter circadian rhythm, later flowering and a red light-sensitive phenotype (Eriksson et al., 2003; Michael et al., 2003; Yamamoto et al., 2003). A recent study showed that CRY2 can interact with the m^6^A writers MTA, MTB and FIP37 and modulate m^6^A writer activity, mRNA methylation and abundance and circadian rhythms in plants (Wang et al., 2021), establishing a preliminary link between light and m^6^A modification. However, to date, there has been very little research on the transcriptome-wide N^6^-methyladenosine methylome profile response to light in soybean or other plants.

Thus, in this study, to further explore whether light affects m^6^A methylation modifications in soybean, we performed MeRIP-seq and RNA-seq analysis on soybean seedlings under dark and continuous light conditions. Here, we obtain the first known m^6^A map of the transcriptome range in soybean. We also compared and analyzed the patterns of m^6^A distribution between dark and continuous light conditions to obtain differentially methylated peaks and then analyzed the potential impacts of m^6^A on gene expression regulation under different light conditions. Collectively, our data constitute a comprehensive picture of m^6^A methylation in mRNA in soybean seedlings and provide a basis for future studies of its function and biological significance in soybean.

## 2 Materials and methods

### 2.1 Plant material and RNA isolation

Soybean varieties TL1 (Tianlong 1) and Williams 82 were grown under continuous light or dark conditions in a growth chamber at 25°C. Aboveground tissue was harvested on day 4 after germination and flash frozen in liquid nitrogen. Total RNA was extracted using TRIzol reagent (Invitrogen, Cat. No 15596026).

### 2.2 High-throughput m^6^A-seq and RNA-seq

Total RNA from each sample was isolated using TRIzol reagent (Invitrogen) and then quantified using a NanoDrop ND-1000 instrument and gel electrophoresis. Polyadenylated RNA was extracted using VAHTS mRNA Capture Beads (VAHTS, Cat. No. N401-01/02), fragmented to 100 nt-200 nt using 20 mM ZnCl_2_ at 95°C for 5 min-10 min and placed on ice. A portion of the purified RNA was kept as input. The remainder of the purified RNA was incubated with m^6^A antibody in IP buffer for 2 h at 4°C. Then, the immunoprecipitation reaction was incubated with protein A/G magnetic beads (Thermo Scientific, Cat. No. 88845) for 2 h at 4°C. The beads were then washed two times with 1× IP buffer, and the RNA was eluted with phenol–chloroform. Then, the sample was dissolved in RNase-free H_2_O. The m^6^A -seq libraries were constructed with a KC-Digital™ stranded mRNA library kit (Seqhealth, Cat. No. DR08502) according to the provided instructions.

RNA-seq libraries were prepared as follows: high-quality mRNA was obtained and fragmented using fragmentation buffer. Using the fragmented mRNA as a template, six-base random primers were used to synthesize single-stranded cDNA, and then buffer, dNTPs and DNA polymerase I were added to synthesize double-stranded cDNA. The purified double-stranded cDNA was eluted for end repair and poly(A) tailing; then, a UID adapter was added as sequencing adapter. Magnetic beads were used to recover the target fragment, and PCR amplification was conducted. Library quality was verified using gel electrophoresis and Qubit 2.0. Finally, the samples were sequenced using an Illumina gene sequencer.

### 2.3 Data analysis

After the data were downloaded, the quality of the reads was evaluated with FastQC (version 0.11.5), and Trimmomatic (version 0.36) was used for data quality control, mainly removal of the linker sequence from the reads and elimination of low-quality reads. Using STAR (version 2.5.3a) software, we mapped the clean data from the input and IP-sequenced samples to the soybean reference genome. Following previous studies, we used RSeQC (version 2.6) to evaluate the mapped read distribution, coverage uniformity, strand specificity and other parameters. Next, based on the background information (input RNA-seq data), we used the R package exomePeak2 (Version 3.8) to obtain robust and high-resolution RIP-Seq peak predictions with two biological replicates. Bedtools (Version v2.25.0) was used to annotate m^6^A peaks in the soybean annotation file, characterize the distribution of peaks on chromosomes, and count the peaks in the transcript regions. The m^6^A site differential algorithm was used for the identification of differentially methylated peaks between the dark and white light samples with criteria of P value <0.05 and log_2_ (fold change) ≥1. The conserved sequences (RRACH and DRACH) of identified m^6^A peaks were found by HOMER (Version v4.10). According to past research, we used deepTools (Version 2.4.1) to study the coverage mapping of reads and peaks in the CDSs and UTRs of all genes and generate a coverage heatmap.

To further explore the essential role of m^6^A mRNA modification in soybean, the identified m^6^A peaks and differentially expressed genes (DEGs) were subjected to network analyses. Here, we used reads per kilobase per million reads (RPKM) as a measure of gene expression levels with a cut off of log2FC ≥ 1 and P value < 0.05 (Mortazavi A. et al., 2008). RPKM was calculated as total exon reads/mapped reads (millions) x exon length (KB). GO and KEGG analyses of the differentially methylated genes and differentially expressed genes were conducted using the Gene Ontology Database (http://www.geneontology.org/) and Kyoto Encyclopedia of Genes and Genomes database (http://www.genome.jp/kegg/). We used Replicate Multivariate Analysis of Transcript Splicing (rMATS) software for differential alternative splicing (AS) analysis. Related data are presented in **Table S1**.

### 2.4 qRT–PCR

The aboveground tissue was quickly frozen with liquid nitrogen and ground into a powder with a mortar. Total RNA was extracted using TRIzol reagent. First-strand cDNA was synthesized from 3 μg of total RNA in a 20 μL volume using an Oligo(dT)_18_ primer with TransScript II One-Step gDNA Removal (TransGen Biotech. Cat. No. AT311-03) and cDNA Synthesis SuperMix time PCR was performed on Biometra Tone 96 G according to a two-step method using the ChamQ Universal SYBR qPCR Master kit (Vazyme. Cat. No. Q711-02). Briefly, each 10 μL qRT–PCR contained 4.6 μL of 20-fold diluted cDNA, 0.2 μL of each primer and 5 μL ChamQ Universal SYBR Qpcr Master Mix. The reaction procedure was predenaturated at 95 °C for 30s, followed by a 40-cycle program (95°C 10 s and 60°C 30s). *GmActin* (*Glyma.18G290800*) was used as an internal control to calculate the transcript levels of selected genes, which were quantified by the cycle threshold 2^-ΔCt^ method. △Ct=Ct (selected gene)-Ct (*GmActin*). Each set of experiments was performed with at least three biological replicates and two technical replicates. Related genes and primer lists are presented in **Table S1**.

### 2.5 m^6^A IP-qPCR

60 μg of total RNA per sample was used, fragmented with 0.272 g ZnCl2 (0.1 M) at 94°C for 100 s and then incubated with beads for at least 3 h or overnight. 30 μL of fragmented mRNA was used as the input control. m^6^A-IP was performed using the magna RIP kit (Millipore, 17-700). 50 μL of beads was used and then immunoprecipitated with 5 μg anti-m^6^A antibody (Synaptic Systems, 202003) at room temperature for 30 min-1 h. Reverse transcription was performed with random primers or specific primers. PCR amplification was performed by qRT-PCR. Each set of experiments was performed with at least three biological replicates and three technical replicates.

## 3 Results

### 3.1 m^6^A methylation is common and conserved in soybean

**Figure 1:**
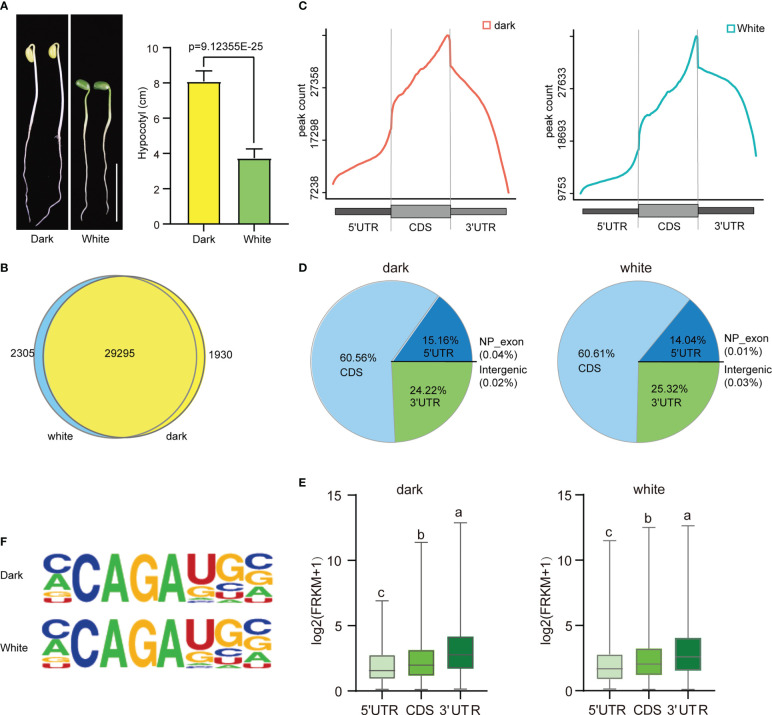
Overview of the m^6^A methylome in soybean. **(A)** The phenotype and hypocotyl statistics of Williams 82 grown in the dark and with continuous white light (100 μmol m^-2^ s^-1^) for 4 days. Scale bars correspond to 5 cm.The comparisons were performed using Student’s t-tests. Data are shown as means ± SD (n = 15). **(B)** Venn diagram showing the overlap of m^6^A peak-containing genes in two independent m^6^A-seq experiments for wild type plants grown under dark and continuous white light conditions. **(C)** Distribution of m^6^A peaks in transcript segments. **(D)** Pie charts showing the distribution of m^6^A peaks plants grown under dark and continuous white light conditions. **(E)** Gene expression (log2(FRKM+1)) of m^6^A peaks within three transcript segments. **(F)** Most representative motifs called for each group (dark and white light). The letters above the bars indicate significant differences (P < 0.05) as determined by one-way ANOVA.

The effect of light on soybean germination and growth were has been studied extensively. Our study showed that soybean seedlings grown in continuous dark conditions had a slightly lower germination percentage than those grown under continuous white light (96.18% vs. 100%) (**Supplementary Figure 1A**). However, the hypocotyl length (8.085 cm vs. 3.74 cm) (**Figure 1A**) and root length (11.4 cm vs. 9.475 cm) (**Supplementary Figure 1B**) of the seedlings grown in the dark were significantly higher than those grown in continuous white light. These results suggest that continuous dark conditions may be beneficial for soybean seedling growth in terms of hypocotyl and root length. To further investigate the physiological differences between these two growth conditions, we performed m6A MeRIP-seq of aboveground tissue on four-day soybean seedlings subjected to continuous dark and continuous light growth conditions. After quality control, high-quality sequencing data were uniquely mapped into the soybean reference genome using STAR (version 2.5.3a) software (**Supplementary Figure 2**). A total of 123386 m6A peaks with an average length of 200 bp were detected in the total set of soybean samples, of which 61737 m6A enrichment peaks represented the transcripts of 31225 genes expressed in the dark conditions, and 61646 m6A enrichment peaks represented 31600 genes expressed under continuous light. Among them, 29295 genes were detected under both dark and continuous light conditions (**Figure 1B**). The soybean genome (wm82.a4. v1) contains 52872 protein-coding genes, of which 33530 exhibit m6A methylation, indicating that m6A methylation modification is very common in this plant. Our results suggest that m6A methylation is an important factor in regulating the physiological response of soybean seedlings to different light conditions.

Next, we analyzed the distribution of m^6^A peaks along the 5’UTR, CDS and 3’UTR of genes and found that the m^6^A peaks were enriched around the stop codon in all the samples (**Figure 1C** and **Supplementary Figure 3**), similar to the distributions observed in other animals and plants. Intriguingly, we found that there was a slight difference in the abundance of m^6^A peaks at different positions under light and dark conditions, with a slight increase within 5′UTRs (15.16% vs. 14.04%) and a slight decrease within CDSs (60.56% vs. 60.61%) and 3′UTRs (24.22% vs. 25.32%) (**Figure 1D**).

To study the effect of the distribution of the m^6^A peak on gene expression, we analyzed the expression levels of genes containing only one peak, and the results indicated that m^6^A peaks in the 3’UTR correlated with the highest levels of gene expression, followed by peaks in the CDS and 5’UTR (**Figure 1E**).

To determine whether the m^6^A peaks share the common sequence elements previously characterized in plants, we used homer to search for consensus motifs in the dark and continuous light groups. Conserved motifs, such as CCAGAUGC (dark: p=1e-157; white: p=1e-143) were all enriched in both groups (**Figure 1F**). Thus, the same sequence motif observed in mammalian and yeast mRNA appears to be necessary for m^6^A methylation in plant mRNA.

We also analyzed the distribution of m^6^A on chromosomes and revealed a lower density of m^6^A peaks at the centromere regions than at other regions (**Supplementary Figure 4**). Further analysis showed that the average density was the highest on chromosome 13 under both conditions (**Supplementary Figure 4**).

### 3.2 m^6^A methylation is involved in the regulation of alternative splicing

**Figure 2:**
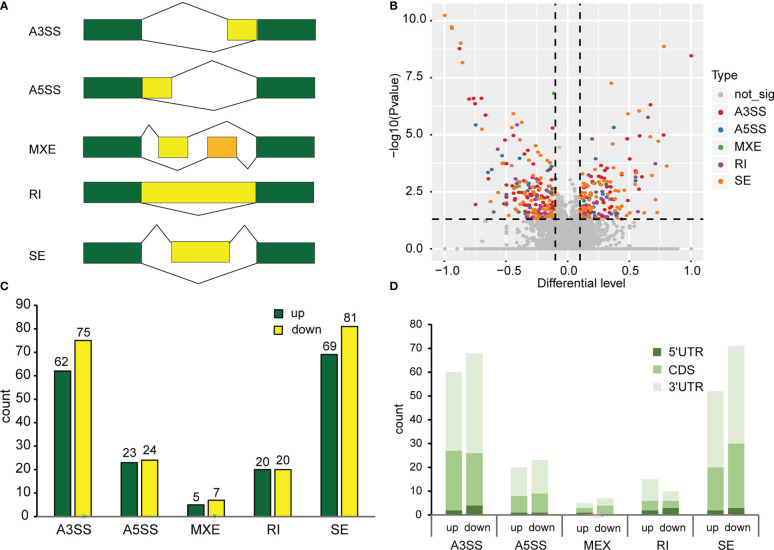
Alternative splicing events in methylated genes. **(A)** Types of alternative splicing events. A3SS, Alternative 3’ splice site; A5SS, Alternative 5’ splice site; MXE, Mutually exclusive exon; RI, Retained intron; SE, Skipped exon. Green boxes represent constitutive exons. Yellow boxes and orange boxes represent alternatively spliced exons. The broken lines represent the way exons are connected. **(B)** Analysis of differential alternative splicing. p value<0.05, |Δψ|>0.05. **(C)** Number of different types of alternative splicing events. **(D)** The effect of methylation of different transcript regions on alternative splicing.

For AS analysis, we used replicate multivariate analysis of transcript splicing (rMATS) software for differential AS analysis. AS events (ASE) can be grouped into five categories, namely, alternative 3’ splice site (A3SS), alternative 5’ splice site (A5SS), mutually exclusive exon (MXE), skipped exon (SE) and retained intron (RI) (**Figure 2A**). There are two situations in which reads are aligned to exons: reads on target and junction reads. We calculated the expression level of alternatively spliced transcripts based on the number of junction reads. We screened ASEs with the criteria p value<0.05 and |Δψ|>0.05. The results revealed that 386 differentially spliced genes were detected, with 179 genes were up-regulated and 207 genes down-regulated when exposed to continuous white light compared to dark conditions (**Figure 2B**). Furthermore, the analysis demonstrated that A3SS and SE were the most frequent alternative splicing events, followed by A5SS, RI, and MXE (**Figure 2C**). A comparison of the number of genes up-regulated and down-regulated under each splicing event, A3SS (62 vs.75), A5SS (23 vs. 24), MEX (5 vs. 7), RI (20 vs. 20) and SE (69 vs. 81), revealed that the number of genes down-regulated was slightly higher than those up-regulated (**Figure 2C**). Moreover, the analysis of genes containing only one m6A peak indicated that an m6A peak in the 3’UTR had the greatest effect on alternative splicing in both dark and continuous white light conditions, followed by peaks in the CDS and 5’UTR (**Figure 2D**). These results suggest that m6A methylation in different transcript regions may affect alternative mRNA splicing in soybean.

### 3.3 Methylated mRNAs are involved in important signaling pathways

**Figure 3:**
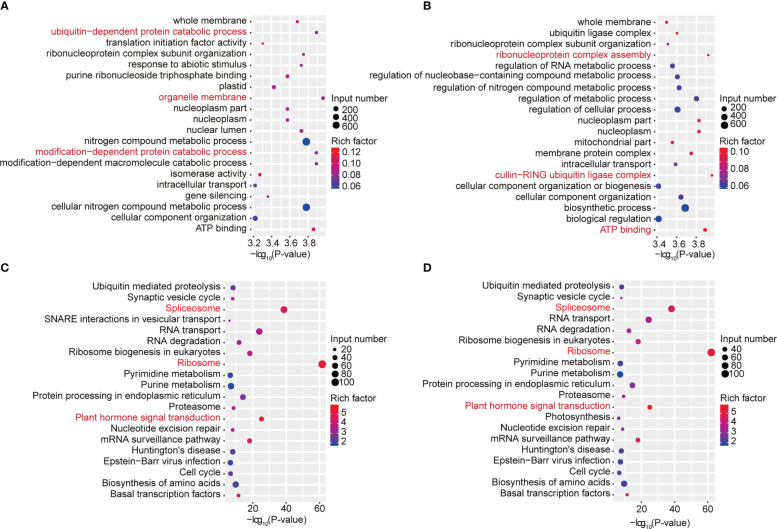
GO and KEGG pathway analyses of all methylated mRNAs. **(A)** The top 20 GO terms among genes modified by m^6^A in the dark. **(B)** The top 20 GO terms among genes modified by m^6^A under continuous white light. **(C)** The top 20 KEGG pathways among genes modified by m^6^A in the dark. **(D)** The top 20 KEGG pathways among genes modified by m^6^A under continuous white light.

To study the biological significance of m^6^A modification in soybean, GO and KEGG pathway analyses of the mRNAs methylated in dark and continuous light conditions were conducted with the p value <0.05 and log_2_ fold change ≥1. Gene Ontology (GO) enrichment analysis of the genes methylated in the dark showed that “ubiquitin-dependent protein catabolic process”, “organelle membrane” and “modification-dependent protein catabolic process” terms were enriched (**Figure 3A**). However, Gene Ontology (GO) enrichment analysis of the genes methylated under continuous light showed that “ribonucleoprotein complex assembly”, “cullin-RING ubiquitin ligase complex” and “ATP binding” terms were enriched (**Figure 3B**). For KEGG pathway analysis, genes methylated in dark and continuous light were both enriched in the “spliceosome”, “ribosome” and “plant hormone signal transduction” pathways (**Figures 3C, D**).

### 3.4 Conjoint analysis of MeRIP-seq and RNA-seq data

**Figure 4:**
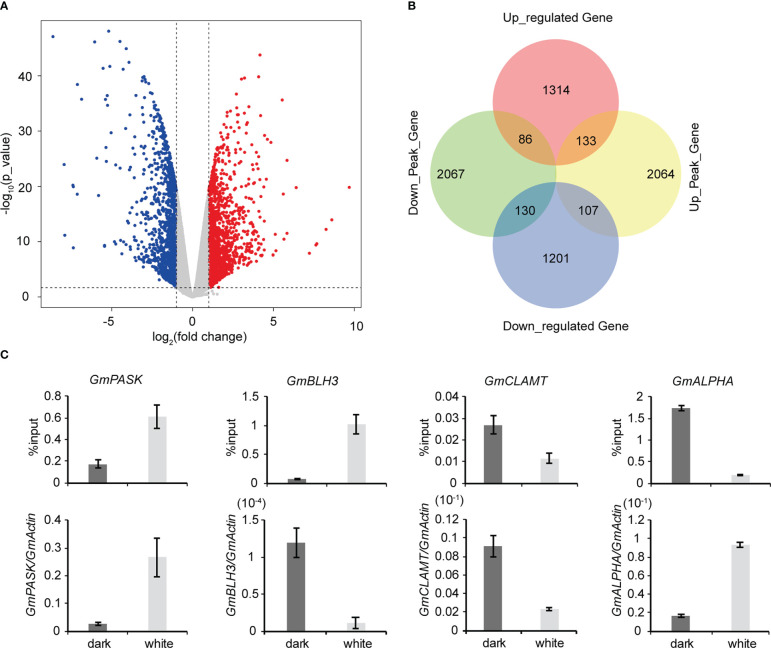
Conjoint analysis of MeRIP-seq and RNA-seq data. **(A)** Scatter plots showing the differential expression of genes under dark and continuous white light conditions. |logFC|>1, FC= RPKM (dark)/RPKM (white), p value<0.05. **(B)** Venn diagram showing the distribution of genes with significant changes in both the m^6^A peaks and mRNA levels. **(C)** Validation of differential m^6^A modification and differential gene expression. The values represent the means ± SD (n=3).

To explore the roles of m^6^A methylation in the soybean response to light, we compared the modification profiles of seedlings grown in the dark and under continuous light treatment. Using RNA-seq, we characterized the transcriptome profiles of soybean seedlings from the two groups. Differentially expressed genes were detected. Compared to samples grown under continuous light, samples grown in the dark had 1,533 significantly upregulated genes and 1,438 significantly downregulated genes (**Figures 4A, B**). Gene Ontology (GO) enrichment analysis of upregulated genes revealed that terms related to “RNA biosynthetic process”, “nucleic acid-templated transcription” and “biological regulation” were significantly enriched (**Supplementary Figure 5A**). Similarly, GO enrichment analysis of downregulated genes demonstrated that terms related to “single-organism metabolic process”, “single-organism biosynthetic process” and “catalytic activity” were significantly enriched (**Supplementary Figure 5B**). KEGG pathway analysis revealed that upregulated genes were enriched in “Plant hormone signal transduction”, “Phenylpropanoid biosynthesis” and “Biosynthesis of secondary metabolites” pathways (**Supplementary Figure 5C**), while downregulated genes were enriched in “Photosynthesis”, “Photosynthesis - antenna proteins” and “Carbon fixation in photosynthetic organisms” pathways (**Supplementary Figure 5D**). Next, using MeRIP-seq analysis, we found 1,211 upregulated m^6^A peak-related genes and 3569 downregulated m^6^A peak-related genes. We then performed codifferential analysis to further associate m^6^A modification and gene expression. As a result, all differentially methylated m^6^A peaks with differential mRNA levels (418) were identified by conjoint analysis of the MeRIP-seq and RNA-seq data. Finally, we found that 111 genes exhibited both a significant upregulation of m^6^A and a significant upregulation of expression, 29 genes exhibited both a significant upregulation of m^6^A and a significantly downregulation of expression, 90 genes exhibited both a significant downregulation of m^6^A and a significant upregulation of expression, and 188 differential genes exhibited both a significant downregulation of m^6^A and a significant downregulation of expression (**Figure 4B**). We randomly selected a number of these 418 genes and used m^6^A IP qPCR and qRT–PCR assays to detect their methylation modification levels and transcription levels, respectively (**Figure 4C**; **Supplementary Figure 6**). The results were consistent with the MeRIP-seq and RNA-seq results, further verifying the accuracy of the sequencing results. We also examined the changes in the methylation modification levels and transcription levels of several of these genes in Tianlong 1, another soybean cultivar, and the results were consistent with the sequencing results (**Supplementary Figure 7**), thus verifying the conservation of m^6^A methylation modification among soybean varieties.

### 3.5 Differential mRNAs with differential m^6^A peaks participate in important signaling pathways

**Figure 5:**
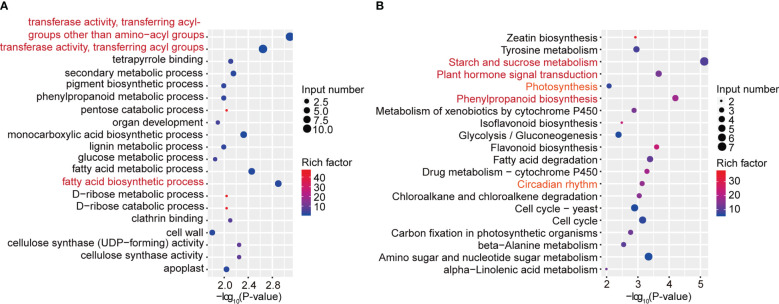
GO and KEGG pathway analyses of differentially expressed genes with differential m^6^A peaks. **(A)** The top 20 GO terms among differentially expressed genes with differentially m^6^A peaks. **(B)** The top 20 KEGG pathways among differentially expressed genes with differentially m^6^A peaks.

To further characterize the important pathways involved in differentially expressed genes with differential m^6^A peaks, we performed GO and KEGG pathway analyses to investigate the biological significance of those 418 genes with synchronous differential expression and differential m^6^A peaks. Gene Ontology (GO) enrichment analysis showed that the biological processes “transferase activity, transferring acyl-groups other than amino-acyl groups”, “transferase activity, transferring acyl groups” and “fatty acid biosynthetic process” were significantly enriched (**Figure 5A**). Moreover, KEGG pathway analysis revealed that in addition to “starch and sucrose metabolism”, “plant hormone signal transduction” and “phenylpropanoid biosynthesis”, the three most significant pathways, the “photosynthesis” and “circadian rhythm” pathways were also associated with differentially expressed genes with differential m^6^A peaks (**Figure 5B**). Next, we used qRT–PCR assays to detect related genes involved in circadian rhythm (**Supplementary Figure 8A**), plant hormone signal transduction (**Supplementary Figure 8B**) and photosynthesis (**Supplementary Figure 8C**) pathways. The results indicated that were consistent with the RNA-seq results. Taken together, these results suggested that differentially methylated m^6^A peaks with differential mRNA levels were involved in important signaling pathways.

### 3.6 Methylation and transcription changes in core light signaling pathway genes

**Figure 6:**
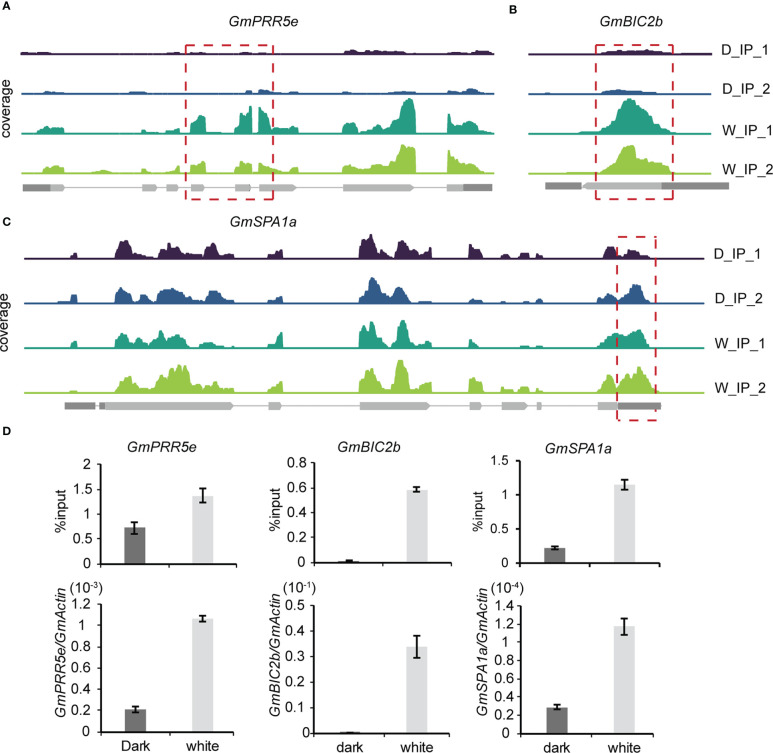
Gene expression-regulated m^6^A modification correlates with light signaling. **(A–C)**, Genomic visualization of m^6^A density maps of representative genes of the light signaling pathway (GmPRR5e, GmBIC2b and GmSPA1a). **(D)** The m^6^A abundance of individual sites of selected genes and their expression levels were analyzed by m^6^A-IP-qPCR and RT–qPCR. The values represent the means ± SD (n=3).

To further explore the effect of m^6^A methylation on the soybean growth response to light, methylation modification levels and transcription levels of core genes in the light signaling pathway were detected and compared between the light and dark groups. Among the 418 differentially expressed genes with differential m^6^A peaks, we found three core light signaling pathway genes, namely, *GmPRR5e* (*Glyma.19G260400*), *GmBIC2b* (*Glyma.13G31*6500) and *GmSPA1a* (*Glyma.16G027200*). *GmPRR5e* and *GmBIC2b* were upregulated in the light group and exhibited an m^6^A peak in their CDS regions (**Figures 6A, B**). *GmSPA1a* was also upregulated in the light group but the m^6^A peak was observed in its 3’UTR region (**Figure 6C**). To validate these findings, we used m^6^A IP qPCR and qRT–PCR assays to detect the methylation modification levels and transcription levels of these genes, respectively (**Figure 6D**). The results were consistent with the sequencing results, indicating that the transcription levels of these three genes were significantly increased in response to light. Therefore, the changes in the m^6^A levels and expression levels of light signaling pathway-related genes in soybean under light conditions may contribute to the phenotypic differences observed in soybean in response to light. Our findings suggest that light affects the levels of methylation modification in soybean seedlings, and that changes in the levels of methylation modification may be essential for soybean growth.

## 4 Discussion

Recent studies have demonstrated that m^6^A writers, erasers and readers are essential for normal plant development and are involved in the plant response to abiotic stresses (Zhong et al., 2008; Bodi et al., 2012; Hu et al., 2019). However, the role of m^6^A methylation in light mediated responses in soybean remains largely unexplored. While it is known that light and circadian cycles can affect the stability and translation of specific plant transcripts (Tang et al., 2003; Juntawong and Bailey-Serres, 2012), further research is needed to investigate the effects of light and circadian rhythm on soybean m^6^A methylation and its associated mechanisms.

In this study, we used soybean as a material to investigate the dynamic modification of m^6^A under light and dark conditions. Our findings showed that the distribution characteristics of m^6^A in soybean are similar to those observed in Arabidopsis and other plants, with m^6^A being significantly enriched at stop codons and 3’UTRs, and the conserved CCAGAUGC motifs being found in yeast, plants, and mammals (Dominissini et al., 2012; Schwartz et al., 2013). Furthermore, we observed that the genes with m^6^A modifications in the 3’UTR had higher expression levels, which is contrary to the findings in mammalian systems, where m^6^A modification around stop codons and at 3’UTRs is negatively correlated with gene expression (Liu et al., 2014; Wang X. et al., 2014; Wang Y. et al., 2014). In addition, we found that in soybean, the genes with m^6^A in the 3’UTR are more prone to alternative splicing. Studies in animals have demonstrated that YTHDC1/SRSF3 and SRSF10 differentially regulate AS in a m^6^A-dependent manner, and the binding regions of YTHDC1, SRSF3 and SRSF10 were significantly enriched in coding sequences and 3’UTRs, concurrent with the m^6^A distribution pattern (Adhikari et al., 2016). This indicates that the role of m^6^A modification in plants may be distinct from that in animals.

Soybean is a photoperiod-sensitive crop, which means it is essential to investigate the influence of light on soybean growth and development in order to maximize its adaptability and productivity. Studies have demonstrated that photoperiodic responses in soybean are largely modulated by specific photoreceptors, such as phytochromes, cryptochromes, and phototropins, which enable the plant to sense the daylength and respond accordingly (Liu et al., 2016). Several genes in the light signaling pathway have been identified, such as the COP1/SPA complex, which can regulate photomorphogenic responses appropriate for the light environment (Podolec and Ulm, 2018). *BIC1* and *BIC2* have been shown to inhibit cryptochrome function and regulate hypocotyl elongation in Arabidopsis (Wang et al., 2017). *PRR5* has been found to play a crucial role in the regulation of flowering and the circadian clock in plants (Uehara et al., 2019; Nakamichi et al., 2020). Our conjoint analysis of MeRIP-seq and RNA-seq data revealed 418 differentially expressed genes with differential m^6^A peaks, including *GmSPA1a*, *GmPRR5e* and *GmBIC2b*, providing evidence for a link between soybean light signaling and m^6^A methylation. KEGG pathway analysis indicated that these differentially expressed genes with differential m^6^A peaks were involved in the “photosynthesis” and “circadian rhythm” pathways, suggesting that m^6^A methylation may be involved in the regulation of plant photosynthesis and the circadian clock in soybean. This comprehensive map of light-regulated m^6^A modification in soybean lays a solid foundation for further research into the functional role of light on RNA m^6^A modification in soybean.

## Data availability statement

The original contributions presented in the study are included in the article/**Supplementary Material**. Further inquiries can be directed to the corresponding author. The original high-throughput m^6^A-seq and RNA-seq data had been deposited in GSA (https://ngdc.cncb.ac.cn/gsa/). The accession No is PRJCA015274.

## Author contributions

Contributions TZ designed the studies. LZ, YZ, JL, HL, and BL performed the experiments. LZ conducted the bioinformatic analyses. TZ wrote the manuscript. All authors contributed to the article and approved the submitted version.
