# Supplementary material for: N6-methyladenosine mRNA methylation is important for the light response in soybean
Source: Front Plant Sci. 2023 Apr 4;14:1153840. doi: 10.3389/fpls.2023.1153840 (PMC10110966; doi:10.3389/fpls.2023.1153840)
Supplement: Supplementary file 1 [file DataSheet_1.docx]

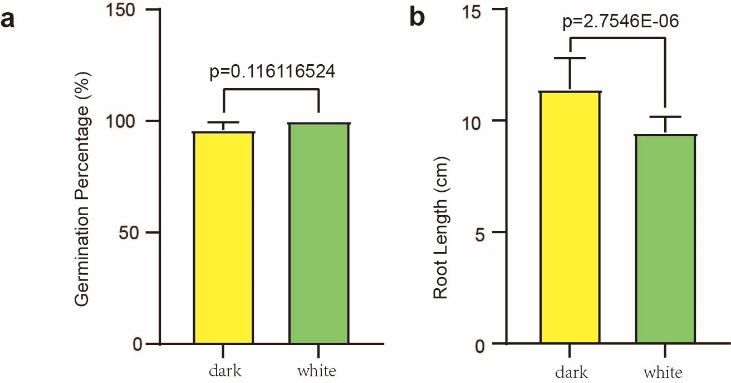


Supplementary Figure 1. Statistical analysis of physiological difference of soybean seedlings grown in dark and continuous white light (100 μmol m^-2^ s^-1^) for 4 days. (a) Comparison of germination percentage (a) and root length (b) between dark and continuous white light conditions. All the above comparisons were performed using Student’s t-tests. Data are shown as means ± SD (n = 15).


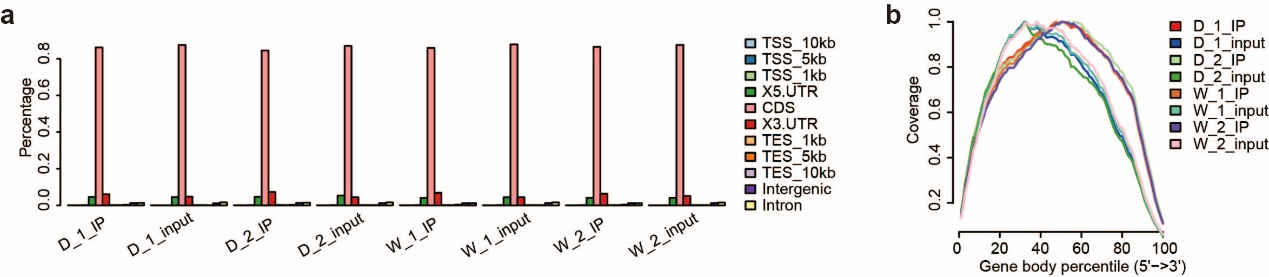
Supplementary Figure 2. The distribution of reads on the reference genome. (a) Histogram of the distribution of reads in different regions of the reference genome. (b) Homogeneity distribution curve of reads on the reference genome.


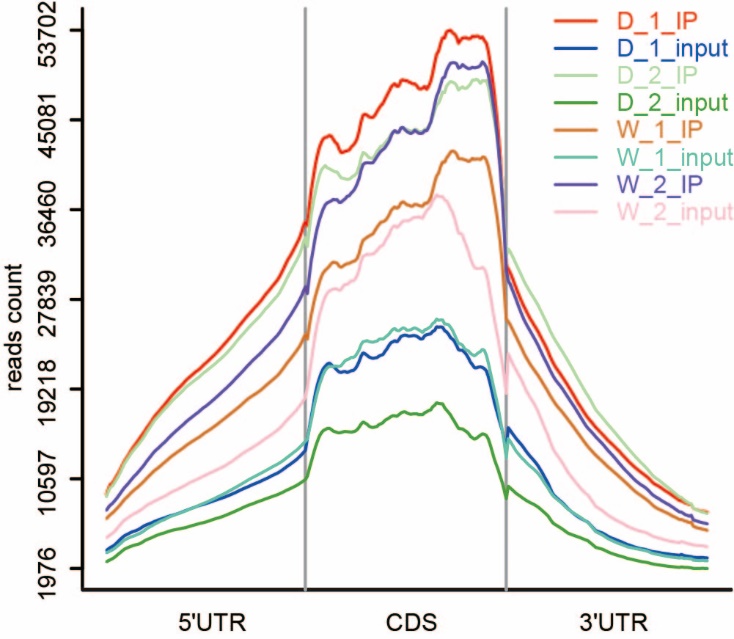


Supplementary Figure 3. The distribution of reads across all genes.


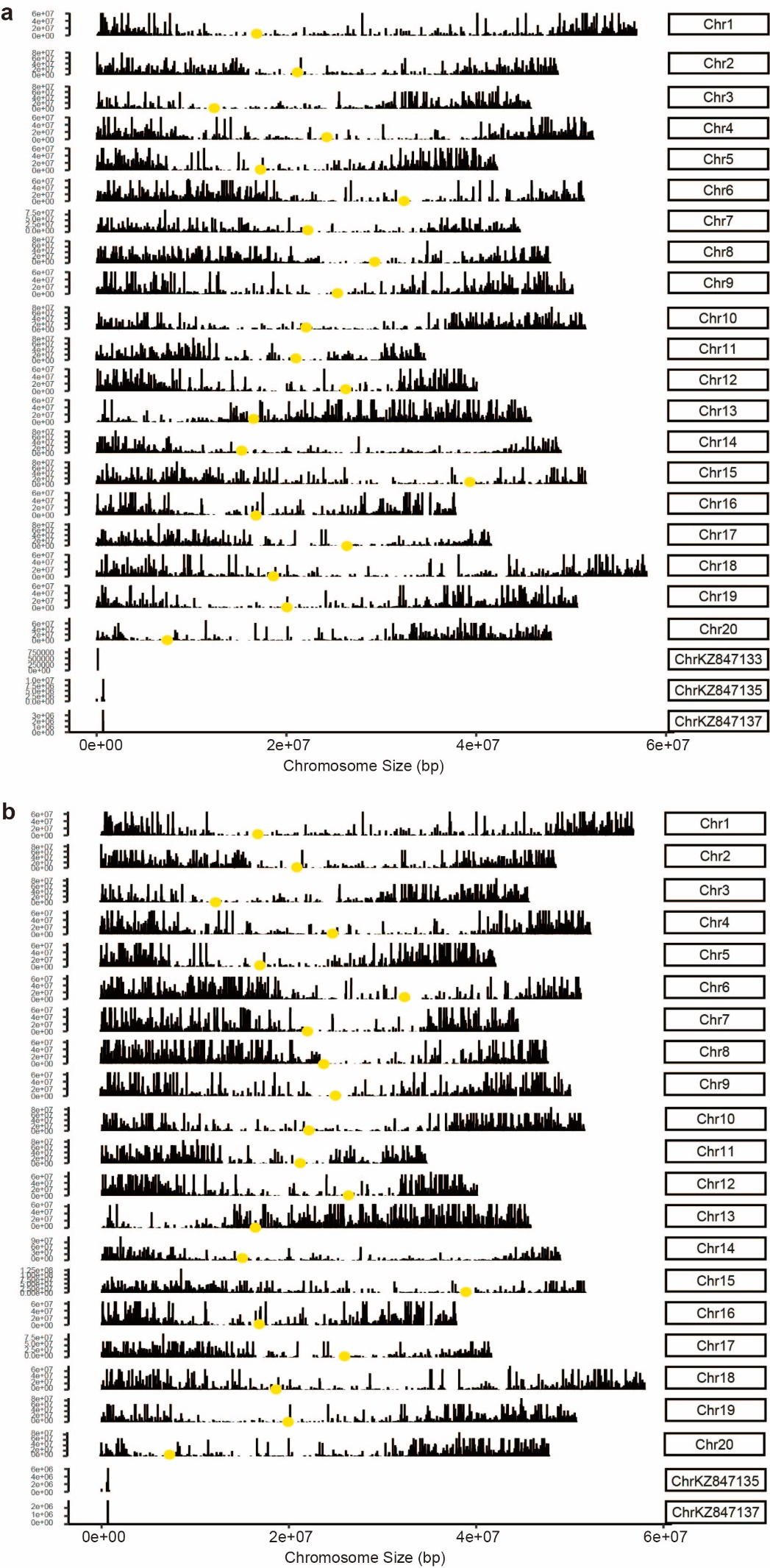


Supplementary Figure 4. Peak distribution on chromosomes. (a) Peak distribution on chromosomes in the dark. (b) Peak distribution on chromosomes in the continuous light. The abscissa represents the length of the chromosome, the right side represents the chromosome number, and the left ordinate represents the peak value of each chromosome. Yellow circles represent centromeres.


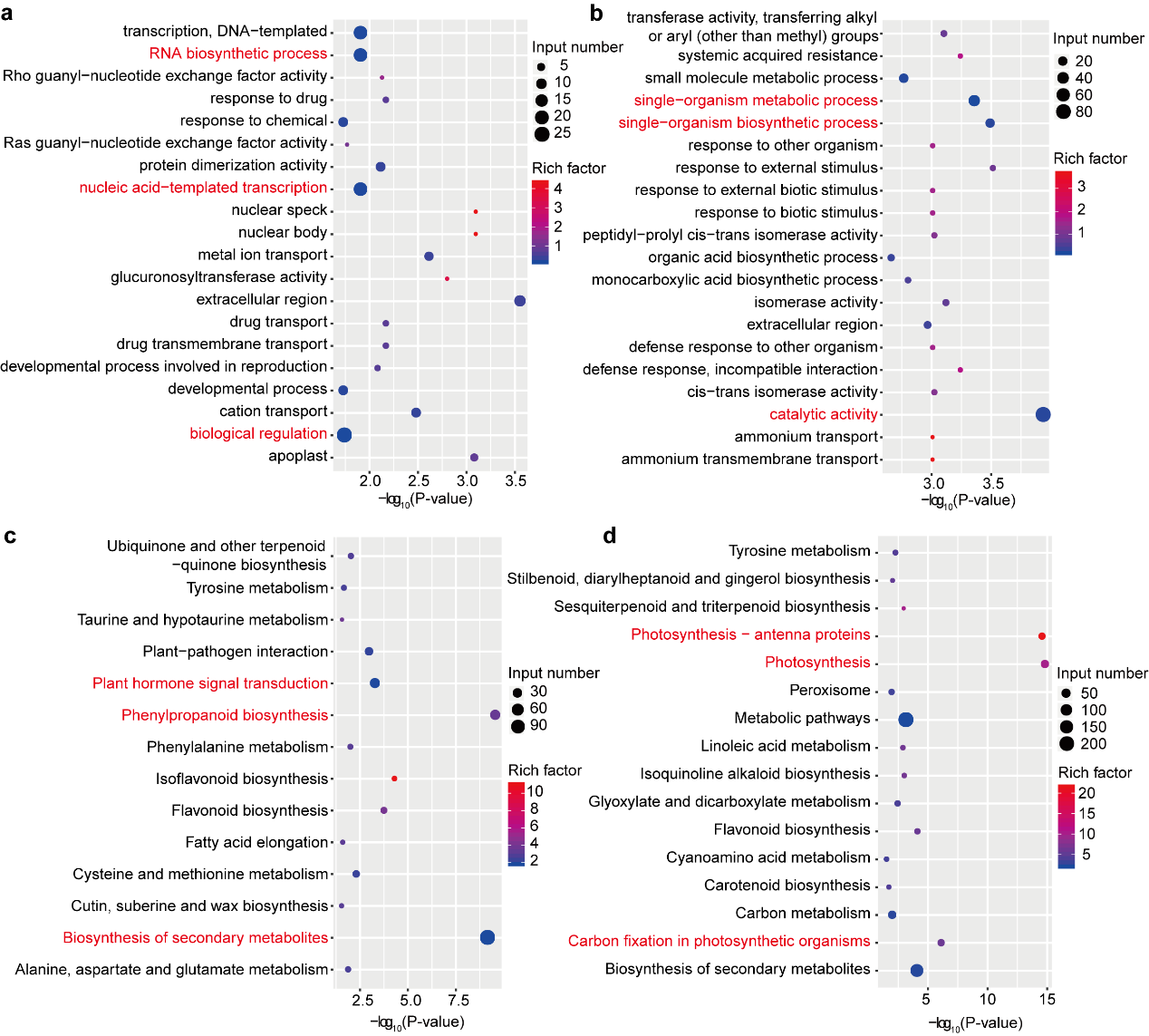


Supplementary Figure 5. GO and KEGG pathway analyses of differentially expressed genes. The top 20 GO terms among upregulated genes (a) and downregulated genes (b). The top 20 KEGG pathways among upregulated genes (c) and downregulated genes (d).


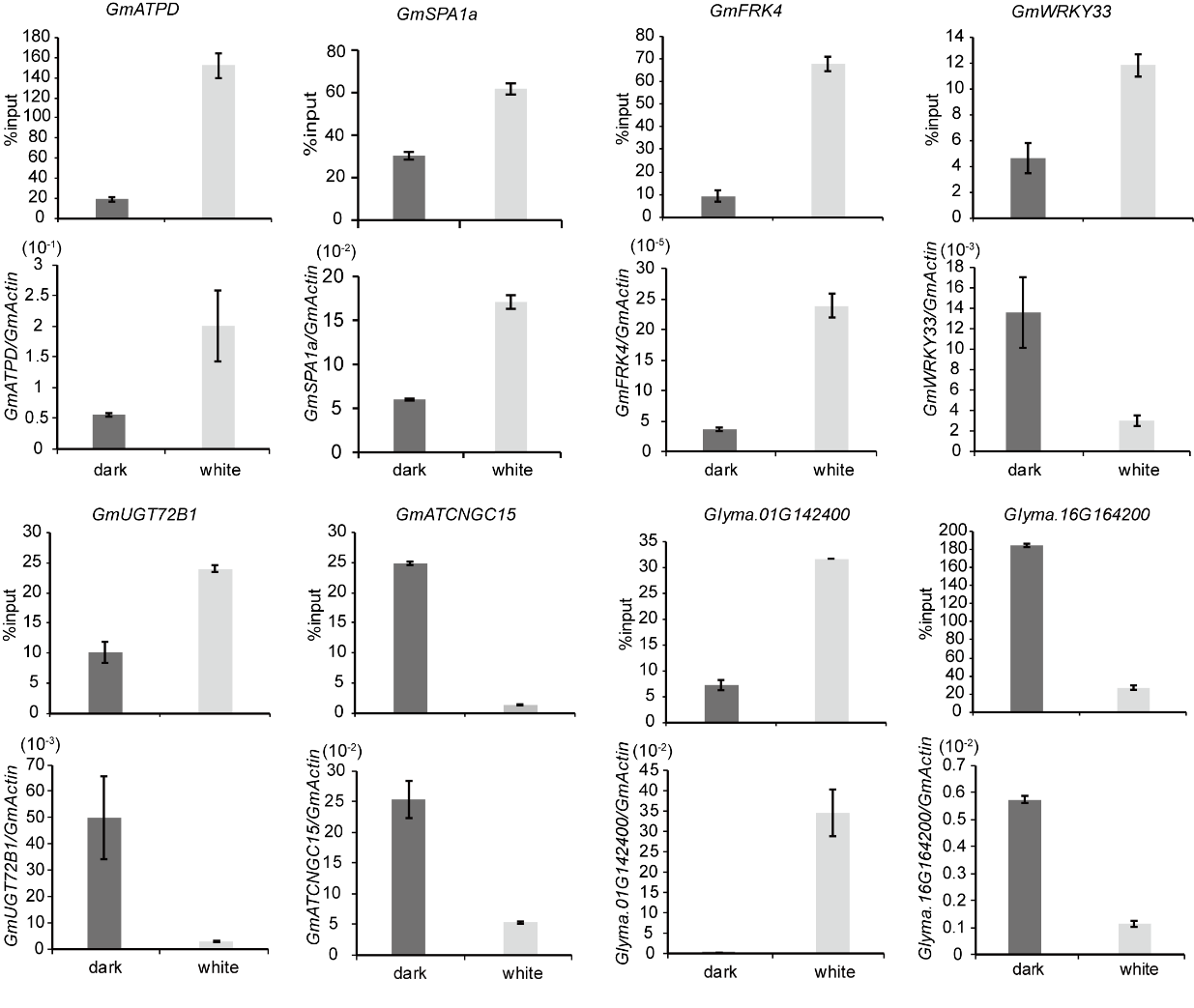


Supplementary Figure 6. Validation of differentially m^6^A peaks related genes and differentially expressed genes in Williams 82. The values represent the means ± SD (n=3).


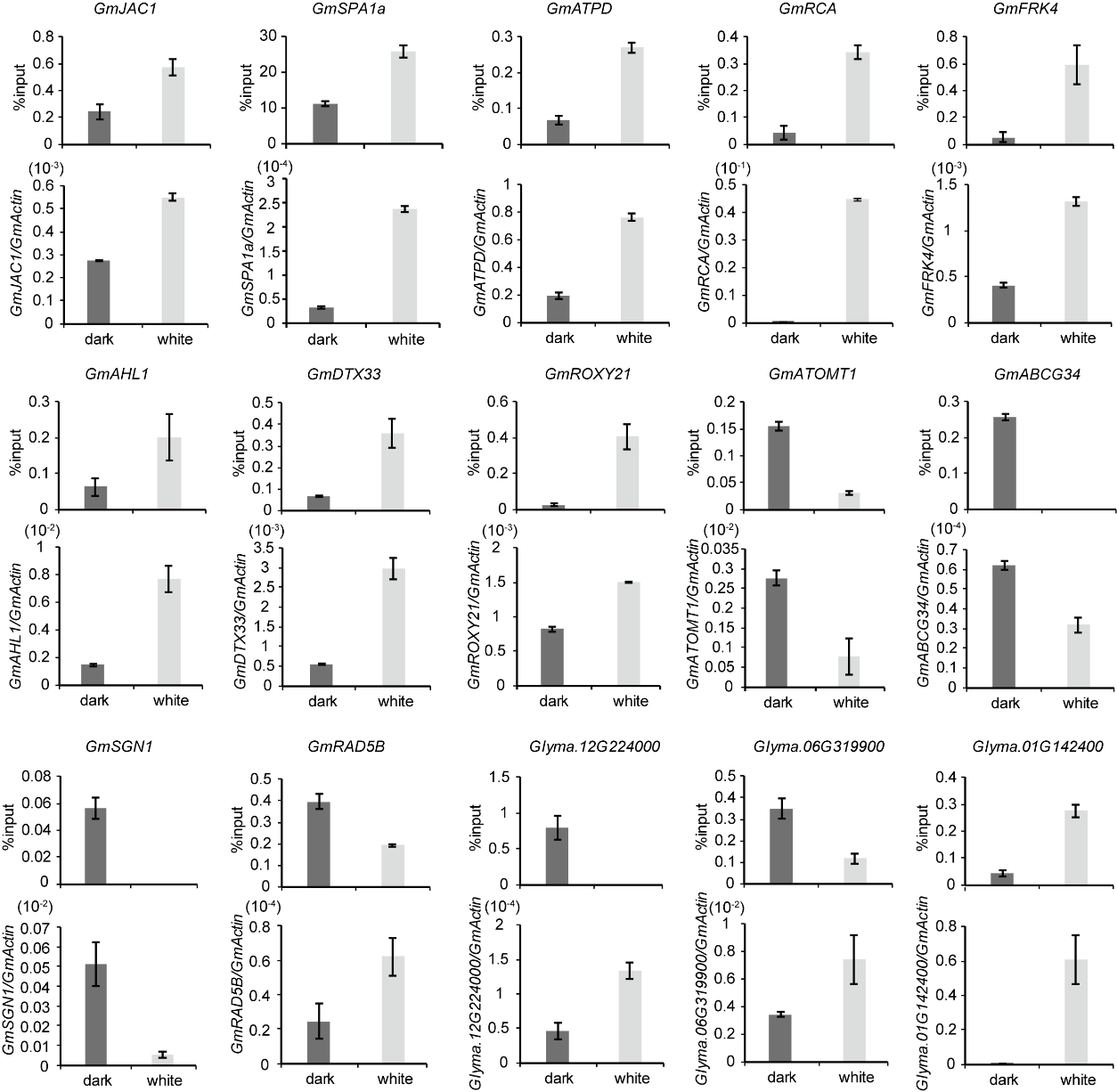


Supplementary Figure 7. Validation of differentially m^6^A peaks related genes and differentially expressed genes in TL1. The values represent the means ± SD (n=3).


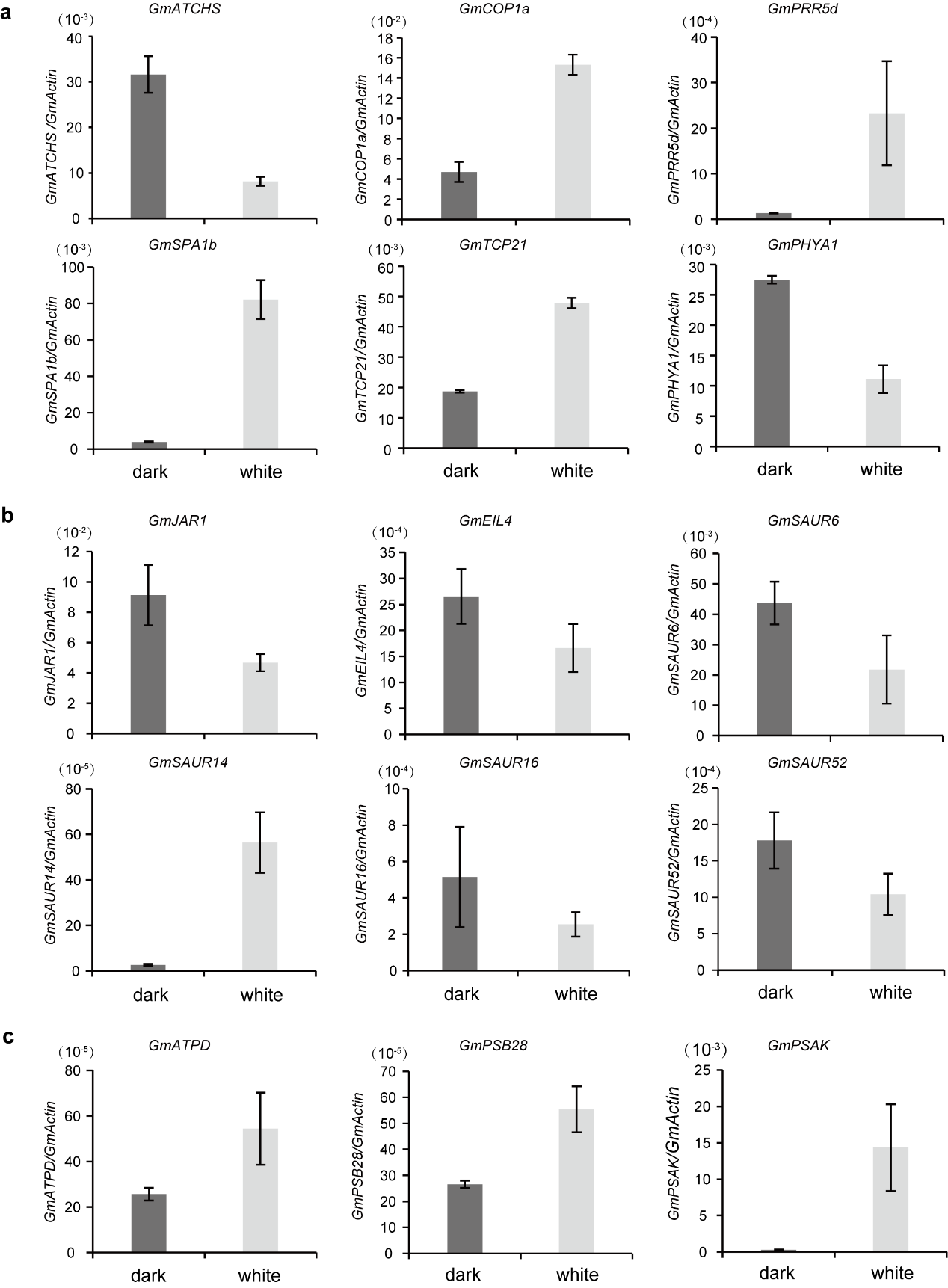


Supplementary Figure 8. Validation of related genes in KEGG pathways. (a) The transcription levels of related genes involved in circadian rhythm pathways, plant hormone signal transduction pathways (b) and photosynthesis pathways (c).The values represent the means ± SD (n=3).
